# Clinical Efficacy Associated with Enhanced Antioxidant Enzyme Activities of Silver Nanoparticles Biosynthesized Using *Moringa oleifera* Leaf Extract, Against Cutaneous Leishmaniasis in a Murine Model of *Leishmania major*

**DOI:** 10.3390/ijerph15051037

**Published:** 2018-05-22

**Authors:** Manal El-khadragy, Ebtesam M. Alolayan, Dina M. Metwally, Mohamed F. Serag El-Din, Sara S. Alobud, Nour I. Alsultan, Sarah S. Alsaif, Manal A. Awad, Ahmed E. Abdel Moneim

**Affiliations:** 1 Chair Vaccines Research of Infectious Diseases, Faculty of Science, King Saud University, Riyadh 11451, Saudi Arabia; eolayan@ksu.edu.sa (E.M.A.); dhasanin@ksu.edu.sa (D.M.M.); saralsaif@ksu.edu.sa (S.S.Als.); 2 Faculty of Science, Zoology Department, King Saud University, Riyadh 11451, Saudi Arabia; 3 Department of Zoology and Entomology, Faculty of Science, Helwan University, Cairo 11795, Egypt; ahmed_abdelmoneim@science.helwan.edu.eg; 4 Parasitology Department, Faculty of Veterinary Medicine, Zagazig University, Zagazig 44519, Egypt; 5 Department of Nutrition and Food Science, Faculty of Home Economics, Minufiya University, Shebin ElKom 32511, Egypt; mserag@ksu.edu.sa; 6 Department of Food Sciences, College of Food and Agricultural Sciences, King Saud University, P.O. Box 2460, Riyadh 11451, Saudi Arabia; 7 Faculty of Medicine, King Saud University, Riyadh 11451, Saudi Arabia; saraal3bud@gmail.com (S.S.Alo.); Nour.alsultan8@gmail.com (N.I.A.); 8 Faculty of Science, Botany and Microbiology Department, King Saud University, Riyadh 11451, Saudi Arabia; 9 King Abdullah Institute for Nanotechnology, King Saud University, Riyadh 11451, Saudi Arabia; mawad@ksu.edu.sa

**Keywords:** cutaneous leishmaniasis, silver nanoparticles, *Moringa oleifera*, antioxidant, gene expression

## Abstract

Leishmaniasis is one of the most significant vector-borne syndromes of individuals. This parasitic infection can be affected by many species of Leishmania, most of which are zoonotic. Natural products have made and are continuing to make important contributions to the search for new antileishmanial agents. The use of plants in the production assembly of silver nanoparticles has drawn attention because of its rapid, eco-friendly, non-pathogenic, economical protocol and provides a single step technique for the biosynthetic process. Hence, we aimed to biosynthesize silver nanoparticles (Ag-NPs) using *Moringa oleifera* leaf extract and investigated the antileishmanial activity of these nanoparticles in a murine model of *Leishmania major* infection. A total of 50 mice were used and divided into five groups—healthy control, infected, infected mice treated with pentostam, infected mice treated with Ag-NPs and infected mice pretreated with Ag-NPs. In the present study, the leaf extract of the plant species *Moringa oleifera* was found to be a good source for the synthesis of silver nanoparticles, their formation being confirmed by color change and stability in solution. In the present murine model of *Leishmania major* infection, we found that oral treatment with silver nanoparticles biosynthesized using *Moringa oleifera* extract resulted in a significant reduction in the average size of leishmaniasis cutaneous lesions compared with untreated mice. Furthermore, the clinical efficacy of *Moringa oleifera* extract was associated with enhanced antioxidant enzyme activities. In conclusion, treatment with silver nanoparticles biosynthesized using *Moringa oleifera* extract has higher and faster clinical efficacy than standard pentavalent antimonial treatment, probably by boosting the endogenous antioxidant activity.

## 1. Introduction

Leishmaniases is one of the most important vector-borne diseases of humans and it is a group of diseases caused by several species of flagellated protozoan parasite belonging to the genus *Leishmania* of the family Trypanosomatidae. At least 20 species of *Leishmania* are known to infect humans, transmitted by female sand flies, Phlebotomus and Lutzomyia *spp.* [1]. Leishmaniasis causes a wide spectrum of clinical manifestations, including skin ulcers and nodules. It can also affect the mucous membranes and may cause disfiguring lesions of the nose. Other species cause damage to the internal organs and cause visceral Leishmaniasis, a life-threatening condition [2].

*Cutaneous leishmaniasis* (CL) occurs almost in all parts of Saudi Arabia [3]. CL lesions usually resolve spontaneously in a few months (without treatment). On the other hand, a disfiguring scar can remain at the infection site due to slow healing and may lead to self-stigma and social stigma and the emergence of negative psychological effects in the young [4]. Lack of proper vaccines, the emergence of drug resistance and the adverse effects of currently used drugs emphasize the need to discover new alternative drugs, particularly from natural products. The toxicity of these agents, their side effects and the growing resistance have become matters of concern. The currently used pentavalent antimony drugs, such as amphotericin B and pentamidine, also have unpleasant side effects. Efforts to find alternative nontoxic, cost effective drugs against Leishmaniasis have led to the development of a few potential drugs from natural resources [5].

Natural products represent a rich source of potential chemical entities for the development of new effective drugs for neglected diseases [6,7]. Scientific evaluation of medicinal plants has made it possible to use some metabolites from quinines, alkaloids, terpenes and flavonoids for the treatment of diseases caused by protozoan parasites [8]. Advantages, such as safety and fewer adverse side effects and low cost have promoted the use of natural extracts as effective anti–leishmanial drugs [7]. Here, we report the efficacy of silver nanoparticles biosynthesized by *Moringa oleifera* extract against a cutaneous leishmaniasis in vivo model.

Synthesis of silver nanoparticles (Ag-NPs) utilizing bacteria, fungi, or plant extracts has emerged as an alternative approach. There are a number of reasons for interest in green biosynthetic methods for Ag-NPs. They are simple, cost-effective, provide large quantities, are harmless and are environmentally friendly [9]. The reduction and stabilization of silver ions is achieved by combining biomolecules such as proteins, amino acids, enzymes, polysaccharides, alkaloids, tannins, phenolics, saponins and vitamins, from plant extracts that are already established as having medicinal value [10]. In addition to microbial organisms, plant materials can be used in the biosynthesis of metallic nanomaterial [11], it has been shown that the plant species of *Ocimum basilicum*, *Azadirachta indica*, *Camellin sinensis*, *Coriandrum sativum*, *Nelumbo nucifera*, curcumin and several other species are good sources for the synthesis of Ag-NPs at a faster rate [12]. Interestingly, biosynthesized Ag-NPs have been reported to show anticancer, antimicrobial, antiinflammatory and antioxidant activities [13]. Furthermore, Allahverdiyev et al. [14] found that Ag-NPs have antileishmanial effects against *L. tropica* parasites by inhibiting growth and inhibiting metabolic activity by impairing mitochondrial function via oxidative stress and the infectivity of promastigotes by preventing survival of amastigotes inside host cells. 

*Moringa oleifera* is a member of Moringaceae that is widely distributed through Southeast Asian countries and Africa. It has long been utilized as a food in some countries due to its high nutritional value and as an Ayurvedic herb for Indian traditional medicine [15]. The leaf extract contains many nutrients including the essential amino acids, vitamins, minerals and β-carotene [16]. *M. oleifera* possesses many pharmacological and biological activities such as hypoglycemic, hypolipidemic, antioxidant, anti-inflammatory and anticancer [17]. In addition, the plant was used against malaria, trypanosomiasis, schistosomiasis and filariasis thus suggesting its inherent antiparasitic activity [18]. Furthermore, Kaur et al. [19] found that the leaf and root extracts of *M. oleifera* showed antileishmanial activity against *L. donavani* with IC_50_ values of 83.0 µg/mL and 47.5 µg/mL, respectively and the authors attributed this activity to the presence of niazinin, a thiocarbamate glycoside.

Hence, we aimed in this study to evaluate the protective effect of silver nanoparticles biosynthesized using *Moringa oleifera* extract against cutaneous lesions induced by *Leishmania major* infection in Balb/c mice.

## 2. Materials and Methods

### 2.1. Plant Material and Extract Preparation

*Moringa oleifera* leaves were purchased from a market located in Riyadh, Saudi Arabia. The plant leaf extract was prepared by mixing 50 g of the leaves with 500 mL distilled water and boiled for 10 min. Then, the obtained fluid was filtrated (Whatman no. 1 filter paper) and the resulting filtrate immediately used for preparing AgNPs.

### 2.2. Total Phenolic 

Total phenolic compound content in moringa methanolic extracts was assayed by the Folin-Ciocalteu method as described previously by Abdel Moneim [20]. Briefly, 0.1 mL of a sample extract was mixed with a volume of 2.5 mL of distilled water in a test tube, followed by the addition of 0.1 mL of undiluted Folin-Ciocalteu reagent (Sigma-Aldrich, St. Louis, MO, USA). The solution was mixed well and then allowed to stand for 6 min before adding 0.5 mL of a 20% sodium carbonate solution. The color was developed for 30 min at room (20 °C) temperature and the absorbance was measured at 760 nm using a spectrophotometer (PD 303 UV spectrophotometer, Apel Co., Limited, Saitama, Japan). A blank sample was prepared using 0.1 mL of methanol instead of extract. The measurement was compared to a calibration curve of gallic acid solutions and expressed as mg equivalent (eq.) gallic acid per g of dry weight extract.

### 2.3. Total Flavonoids

The aluminum chloride colorimetric method was used to determine the total flavonoids content of moringa extract according to Abdel Moneim [20]. In a test tube, 50 µL of the extract mixed with 4 mL of distilled water and then 0.3 mL of 5% NaNO_2_ solution and 0.3 mL of 10% AlCl_3_·6H_2_O were added to the mixture. The mixture was allowed to stand for 6 min and then, 2 mL of 1 mol/L NaOH solution was added and the final volume of the mixture was brought to 10 mL with distilled water. The mixture was allowed to stand for another 15 min and absorbance was measured at 510 nm. The total flavonoid content was calculated from a calibration curve and the result was expressed as mg eq. rutin per g dry weight.

### 2.4. DPPH (2,2–Diphenyl–1–Picrylhydrazyl) Radical Scavenging Activity 

The power of the moringa extract to scavenge DPPH radicals were assayed according to the method of Karakaya and Akillioglu [21], A fresh of 0.08 mM DPPH radical solution in methanol was prepared, A 950 μL of DPPH solution was mixed with 50 μL extract and incubated for 5 min. Exactly 5 min later absorbance readings of mixture was performed at 515 nm (PD 303 UV spectrophotometer, Apel Co., Limited). Antioxidant activity (AA) was expressed as percentage inhibition of DPPH radical by using below equation; AA = 100 − [100 × (A_sample_/A_control_)] where A_sample_ is the absorbance of the sample at t = 5 min and A_control_ is the absorbance of control.

### 2.5. ABTS (2,4,6–Tri(2–Pyridyl)–s–Triazine) Radical Scavenging Activity 

The ABTS^•+^ assay used was determined according to the method of Gouveia and Castilho [22]. The ABTS^•+^ radical solution was prepared by reacting 50 mL of 2 mM ABTS solution with 200 μL of 70 mM potassium persulfate solution. This mixture was stored in the dark for 16 h at room temperature and it was stable in this form for two days. For each analysis, the ABTS^•+^ solution was diluted with pH 7.4 phosphate buffered saline (PBS) solution to an initial absorbance of 0.700 ± 0.021 at 734 nm. This solution was freshly prepared for each set of analysis. To determine the antiradical scavenging activity, an aliquot of 100 μL methanolic solution was mixed with 1.8 mL of ABTS^•+^ solution and the decrease in absorbance, at a 734 nm (PD 303 UV spectrophotometer, Apel Co., Limited, Saitama, Japan), was recorded during 6 min. Results were expressed as μmol Trolox equivalent per g of dried extract (μmol eq. Trolox/g), based on the Trolox calibration curve.

### 2.6. Ferric Reducing Antioxidant Power (FRAP)

Ferric reducing antioxidant power (FRAP) was performed according to the method described by Abdel Moneim [20]. The FRAP reagent included 300 mM acetate buffer, pH 3.6, 10 mM 2,4,6–Tris(2–pyridyl)–s–triazine (TPTZ) in 40 mM HCl and 20 mM FeCl_3_ in the ratio 10:1:1 (*v*/*v*/*v*). Three mL of the FRAP reagent was mixed well with 100 µL of the moringa extract in a test tube and incubated with shacking at 37 °C for 30 min in a water bath. Reduction of ferric–TPTZ to the ferrous complex formed an intense blue color which was measured; at a UV–vis spectrophotometer (PD 303 UV spectrophotometer, Apel Co., Limited) at 593 nm at the end of 4 min. Results were expressed in terms of μmol eq. Trolox per g of dried sample (μmol eq. Trolox/g).

### 2.7. Synthesis of Silver Nanoparticles

Green silver nanoparticles were synthesized by bio reduction of Ag^+^ by using fresh suspension of Moringa extract. 5 mL of the extract was added drop-by-drop to an aqueous solution of AgNO_3_ (50 mL, 0.1 mM/mL) and was stirred at 45–50 °C for 30 min. The ultrasonication was applied to the mixed solution for 3 h. Silver nitrate solution color was changed from colorless solution to deep brown, indicating the formation of Ag-NPs. The residual AgNO_3_ was removed by dialysis against deionized water at 4 °C. The formed Ag-NPs have been analyzed by Zeta sizer (ZEN 3600, Malvern, UK) and characterized using transmission electron microscopy (TEM) (JEM-1011, JEOL, Akishima, Japan). Furthermore, the green silver nanoparticles synthesis was confirmed by UV-Vis spectrophotometer in the range of 200–1000 nm wavelength. The absorption spectra were recorded with Perkin–Elemer Lambda 40 B double beam spectrophotometer using 1 cm matched quartz cells. The stability of Ag-NPs was examined by observing the color of solution after 20, 40, 50 and 60 days of storage in refrigerator at 4 °C.

### 2.8. Leishmania Major and Culture

*L. major* promastigotes of a Saudi sub-strain were used and maintained in RPMI1640 medium (GIBCO, New York, NY, USA) containing fetal bovine serum (FBS) (Sera Laboratories International, Horsted Keynes, UK), 100 U/mL penicillin + 100 mg/mL streptomycin (BioWhittaker, Verviers, Belgium) and 1% L glutamine in 25 mL culture flasks. Each flask was incubated on its side in an incubator set at 25 °C. This incubation method increases the medium aeration, allowing the cells to recover and grow faster. Cultures were passaged after 4 days of incubation. Morphology and motility of promastigotes were observed by using an inverted microscope.

### 2.9. Experimental Protocol

BALB/c female mice (n = 50; eight weeks old) for the in vivo experiment were obtained from the animal house at the Female Center for Scientific and Medical colleges, Riyadh, Saudi Arabia. Mice were challenged with *L. major* by subcutaneous injection of 0.1 mL of RPMI 1640 media containing 10^6^ promastigote. The animals were housed in wire-bottomed cages under standard conditions of illumination with a 12-h light-dark cycle and at a temperature of 25 ± 1 °C for one week until the beginning of treatment. Animals were provided with tap water and a balanced diet *ad libitum*. All experiments were performed in accordance with the requirements of the local animal ethics committee, College of Science (King Saud University). The animals were randomly divided into 5 groups, with 10 mice in each group. The five groups were group I (negative control), group II (positive control), group III (infected and treated with sodium stibogluconate (pentostam, 120 mg/kg intramuscularly injected)), group IV (a curative treatment group treated topically with *M. oleifera* nanosilver given daily, which starting when the ulcerative lesion were appeared and continued for four weeks) and group V (a prophylactic treatment group received orally *M. oleifera* nanosilver (SMO, 0.2 mg/mouse) two weeks before infection and continued for four weeks after infection).

Mortality was checked daily and parasitemia was assessed every other day by observing the appearance of lesions (2–6 weeks post-infection). Each week, the lesion size was measured before and after treatment with vernier calipers in two diameters (a, b). The lesion size was calculated using the following formula [13]:

Lesion Size (LS): a + b/2

Four weeks post–infection, mice were sacrificed and skin at the side of lesion was excised promptly. Skin samples for biochemical and molecular analysis were frozen at −80 °C until processed.

### 2.10. Oxidative Stress

Homogenates of skin were prepared in 50 mM Tris-HCl and 300 mM sucrose to measure lipid peroxidation (LPO) in terms of the amount of malondialdehyde (MDA) formed using thiobarbituric acid (TBA) method [23]. Whereas, Green et al. [24] and Ellman [25] methods were applied to determine the levels of nitrite/nitrate (nitric oxide; NO) and glutathione, respectively.

### 2.11. Enzymatic Antioxidant Status

The prepared homogenates of skin were used in determination of superoxide dismutase (SOD) [26], catalase (CAT) [27], glutathione peroxidase (GPx) [28] and glutathione reductase (GRd) [29].

### 2.12. Real Time PCR

Total RNA was extracted from the skin tissue samples using an RNeasy plus Minikit (Qiagen, Valencia, CA, USA). RNA was reverse transcribed using the RevertAid H minus Reverse Transcriptase (Fermentas, Thermo Fisher Scientific Inc., Waltham, MA, USA). Real time PCR reactions were performed using Applied Biosystems 7500 Instrument. The relative gene expression was determined with power SYBR Green (Life Technologies, Carlsbad, CA, USA) and by the comparative threshold cycle method of Pfaffl [30]. The PCR primers for INOS, GPx and GRd genes were synthesized by Jena Bioscience GmbH (Jena, Germany). Primers were designed using Primer-Blast program from NCBI. mRNA levels for each sample were normalized to *β*-actin. The primer sets used the following:

iNOS (S): 5′–GAAAGAACTCGGGCATACCT–3′.
iNOS (AS): 5′–GGCGAAGAACAATCCACAAC–3′.
GPx (S): 5′–CGGTTTCCCGTGCAATCAGT–3′.
GPx (AS): 5′–ACACCGGGGACCAAATGATG–3′.
GRd (S): 5′–AGCCCACAGCGGAAGTCAAC–3′.
GRd (AS): 5′–CAATGTAACCGGCACCCACA–3′.
*β*–Actin (S): 5′–GGCATCCTGACCCTGAAGTA–3′.
*β*–Actin (AS): 5′–GGGGTGTTGAAGGTCTCAAA–3′.

### 2.13. Determination of Apoptotic Markers in Skin Tissue 

Skin samples homogenates were prepared in lysis buffer and analyzed using a colorimetric caspase-3 assay kit (Sigma-Aldrich Co., Saint Louis, MO, USA), according to the manufacturer’s instructions. Whereas, Bcl-2 protein and Bax protein levels were determined in skin homogenates lysates by ELISA kits (R&D Systems Inc., Minneapolis, MN, USA), the procedure was performed according to instructions of manufacturer. Levels were expressed as ng/mg tissue protein.

### 2.14. Statistical Analysis

Results represent means ± standard errors of the means (SEM). Data were analyzed by one-way analysis of variance (ANOVA). For the comparison of significance between groups, Duncan’s test was used as post hoc test according to the Statistical Package for the Social Sciences (SPSS version 20.0 IBM, Armonk, NY, USA).

## 3. Results

The total amount of the phenolic and flavonoids content present in the investigated extract is shown in Table 1, which was found to be 9.466 ± 0.754 mg eq. gallic acid/g and 0.609 ± 0.026 mg eq. rutin/g, respectively. Furthermore, the results revealed that the extract has potent free radical scavenging power. For the DPPH, ABTS and FRAB assays the values obtained were of 36.88 ± 0.99, 4.989 ± 0.034 and 0.185 ± 0.0005 μmol eq. Trolox/g, respectively.

**Figure 1:**
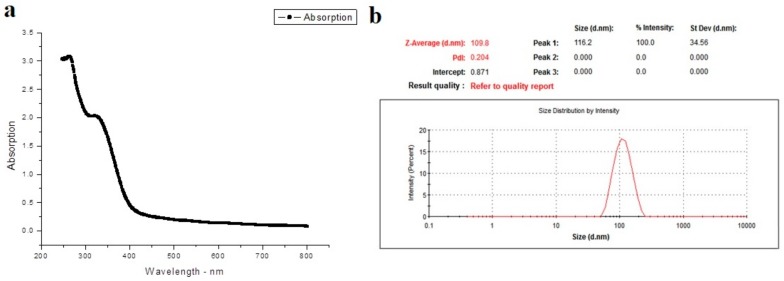
(**a**) The absorption spectrum of the green silver nanoparticles synthesized with *Moringa oleifera* leaf extract; (**b**) Presents a graph of a zeta sizer for measuring the average size of green silver nanoparticles.

**Figure 2:**
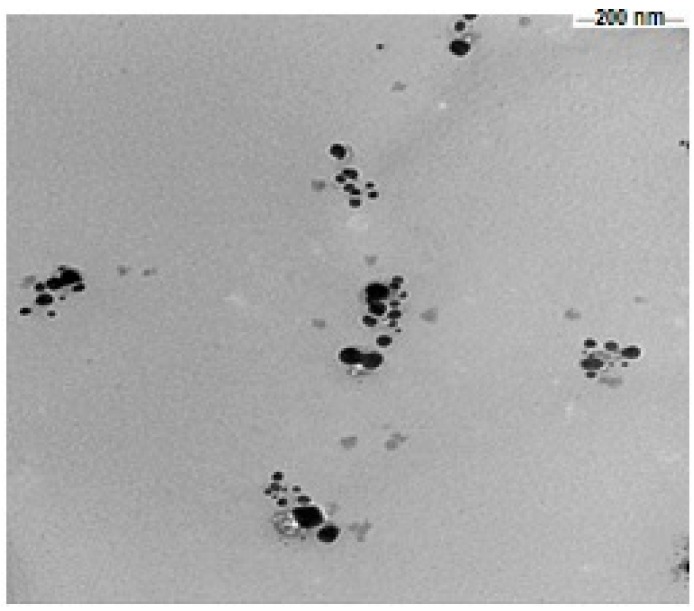
Presents a graph of transition electron microscopy (TEM) image of green silver nanoparticles synthesized (scale bar: 200 nm).

The UV-visible spectrophotometer was used in order to confirm the presence of Ag-NPs. As shown in Figure 1a, the appearance of a band around 330 nm indicates the formation of Ag-NPs particle. In order to determine the size of biosynthesized Ag-NPs particle, Zeta analysis was performed to determine the average particle diameter in nanometer (d.nm). Ag-NPs particle size distribution is shown in Figure 1b. From the current results, it was clear that the size of Ag-NPs particles ranged from 51 to 226 d.nm with average size of 116.2 d.nm. Furthermore, TEM image (Figure 2) demonstrated that the most Ag-NPs were obviously spherical or polygonal in morphology, with size up to 109 nm. Interestingly, after 50 days of storage at 4 °C, the color of Ag-NPs aqueous solution was not change indicating the stability of Ag-NPs particles. However, the color changed into colorless after 60 days.

**Figure 3:**
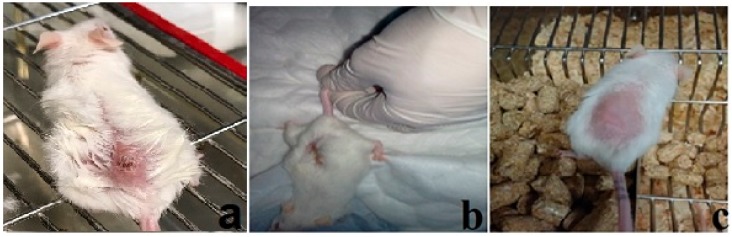
Cutaneous lesions of inoculated infected mouse in untreated group (**a**); Ag-NPs treatment starting when the ulcerative lesion was appeared (**b**) and Ag-NPs treatment starting two weeks before the infection (**c**).

The current experiment was carried out to determine whether concurrent treatment or pretreatment of mice with the silver nanoparticles biosynthesized using *M. oleifera* (SMO) could protect from infection with *L. major*. Clinically, lesions in non–treated infected mice showed redness and swelling at the site of *L. major* inoculation on the 3rd week post infection (Figure 3). 

**Figure 4:**
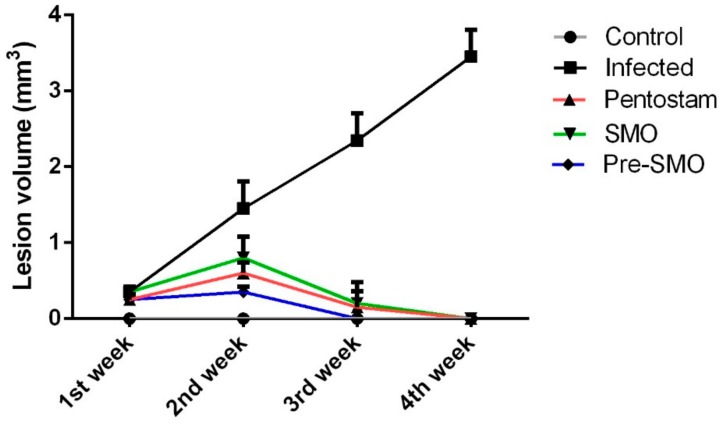
Lesion size in mouse skin one, two, three and four weeks after infection. Lesion sizes, measured with a digital caliper as described in the Material and Methods. Each point represents the mean ± SEM (n = 10).

In the infected control mice, the mean lesion size increased gradually by the 4th week of the experiment. It was observed that lesion size started to decrease gradually after the treatment regimen was initiated and lesions at the 2nd week of the experiment after infection had completely disappeared (Figure 4).

**Figure 5:**
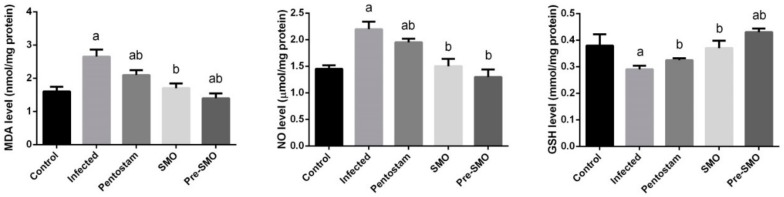
Effect of Ag-NPs biosynthesized by *Moringa oleifera* leaf extract pre-treatment or post-treatment and pentostam on oxidative stress markers (malonaldyhide, nitric oxide and glutathione) of control and experimental groups four weeks after infection. Values are mean ± SEM (n = 10), ^a^ *p* < 0.05, significant change compared to –ve Control group; ^b^ *p* < 0.05, significant change compared to +ve Control group. MDA: malonaldyhide; NO: nitric oxide and GSH: glutathione.

Cutaneous leishmaniasis infection significantly (*p* < 0.05) enhanced the formation of lipid peroxidation and nitric oxide generation whereas, the dermal glutathione content was depleted significantly compared to the control mice. SMO treatment significantly reversed the increased LPO and NO (nitric oxide) and the GSH (glutathione) content was increased significantly indicating the antioxidant property of SMO. Interestingly, SMO pretreatment was more potent than pentostam, a standard drug for cutaneous leishmaniasis (Figure 5). 

**Figure 6:**
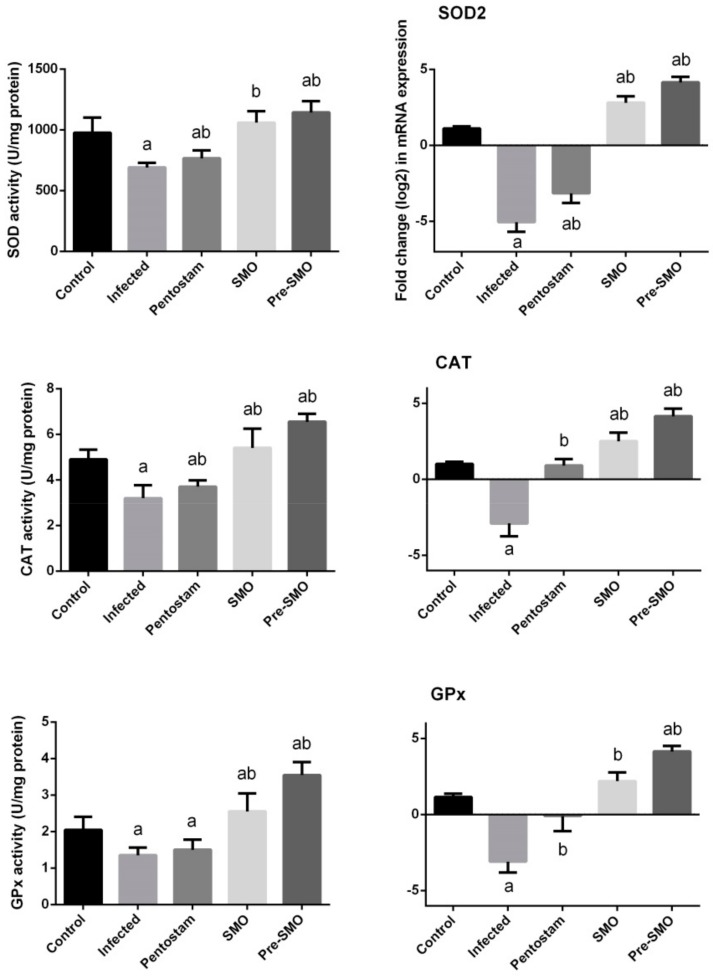
Effect of Ag-NPs biosynthesized by *Moringa oleifera* leaf extract pre-treatment or post-treatment and pentostam on dermal antioxidant enzyme activities (superoxide dismutase, catalase and glutathione peroxidase) of control and experimental groups four weeks after infection. Values are mean ± SEM (n = 10), ^a^ *p* < 0.05, significant change compared to –ve Control group; ^b^ *p* < 0.05, significant change compared to +ve Control group. SOD: superoxide dismutase and CAT: catalase, GPx: Glutathione peroxidase.

Chronic wounds often exhibit oxidative stress that slow down the wound-healing process, suppress collagen deposition and epithelialization and enzymatic antioxidants play crucial role in accelerating wound-healing [31]. Herein, we found that *L. major* infection in the mice inhibited significantly (*p* < 0.05) the antioxidant enzyme activities of SOD, CAT and GPx (Figure 6). Those biochemical findings were confirmed by the molecular analyses. SMO treatment promoted the antioxidant enzymes activity and those enzymes showed higher activities than those in *L. major* infection mice. Furthermore, the results revealed that the pretreatment of SMO led to better findings and pentostam has no antioxidant activity.

**Figure 7:**
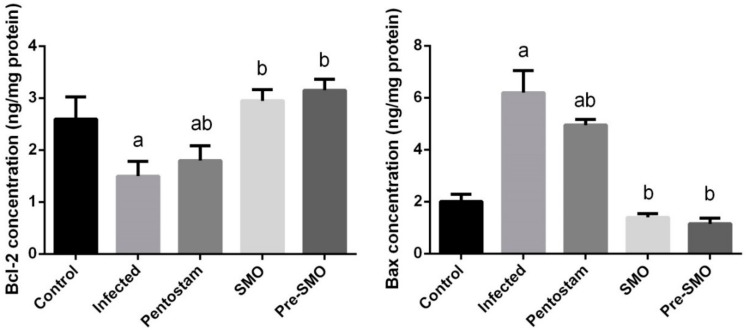
Effect of Ag-NPs biosynthesized *Moringa oleifera* leaf extract pre-treatment or post–treatment and pentostam on dermal pro-apoptotic and anti-apoptotic proteins (Bcl–2 and Bax) in control and experimental groups. Values are mean ± SEM (n = 10), ^a^ *p* < 0.05, significant change compared to –ve Control group; ^b^ *p* < 0.05, significant change compared to +ve Control group. Bax: Bcl–2–associated X protein and Bcl–2: B–cell lymphoma 2.

To investigate whether the observed anti-leishmaniasis effects of SMO were related to the anti-apoptotic activity of SMO, the protein levels of Bcl-2 and Bax in dermal tissue were measured. The current findings revealed that the protein expression of the anti-apoptotic protein Bcl-2 was significantly reduced (*p* < 0.05), while the protein expression level of the pro-apoptotic protein Bax was increased in *L. major* infection mice (Figure 7). However, mice treated with SMO concurrently or prior to *L. major* infection showed significant increase of Bcl-2 and decreased significantly the Bax level. 

## 4. Discussion

In this study, the leaves of *M. oleifera* are good sources for the synthesis of Ag-NPs, the formation of Ag-NPs was confirmed by the color change and stable in the solution. The change in color has a direct correlation with the successful of Ag-NPs formation which may be due to the presence of reducing agents such as terpenoids, flavonoids and polysaccharides [32]. TEM analysis confirmed the range of particle size about 116.2 d.nm have a spherical shape. The carbohydrates, polyphenols and other constituents present in extract of Moringa act as the surface-active stabilizing molecules for the synthesis of Ag-NPs, antioxidative and anti–inflammatory effects. The bio-component of *M. oleifera* extract encapsulates Ag nanoparticles and forms clusters of many numbers of silver nanoparticles. Most of the particles in the TEM images are bound to the bio-organic material. This characters agree with another studies for leaf extract of *M. oleifera* was assessed for the synthesis of Ag-NPs using 1 mM silver nitrate aqueous solution [32].

In the present study, we found that Ag-NPs green synthesized with *M. oleifera* leaf extract treatment caused a significant decline in the average lesion size and complete healing after 14 days compared to standard drug pentostam that needs more than 28 days for complete healing. This is in accordance with a previous report by Oskuee et al. [33].

Reactive oxygen species (ROS) formed during multiple normal processes in tissues and cells which were indicated in the pathogenesis of various parasitic infections including *Leishmania*, *Toxoplasma gondii*, *Giardia lamblia*, *Entameba histolytica* [34,35]. *L. major* induces inflammation by mast cell activation and by producing pro-inflammatory cytokines and mediators. ROS that are generated during an inflammation reaction lead to oxidative damage to non-infected cells. During oxidative damage some free radicals are released that have an important role in collagen damage [36]. In our study, cutaneous leishmaniosis infection impaired the antioxidant system. GSH, the main endogenous antioxidants, was depleted associated with lipid peroxidation, a marker of cellular oxidative stress and have long been recognized as a major consecutive factor of oxidative damage in different diseases. In the present study, elevation in MDA, the end product of lipid peroxidation in infected and pentostam groups were observed. The antioxidant property of *M. oleifera* was recently reported by Tang et al. who found that the leaf extract possessed hydrogen peroxide and hydroxyl radical scavenging activities [37]. However, Ribeiro et al. [38] reported that Ag-NPs showed toxicity in cells by generating ROS. Hence, our results indicated that the biosynthesized of Ag-NPs using *M. oleifera* has a benefit effect on Ag nanoparticles and minimizes its toxicity. 

Lipid peroxidation has been traditionally thought to be the major effect of free radicals and impair the physicochemical properties, fluidity, integrity of cell membrane and apoptosis [39]. The increase in the GSH content well-established with herbal, which was able to reduce the formation of intracellular ROS in response to different pro-oxidant stimuli [40]. The present data suggest that *M. oleifera* is capable to protect cells by stabilizing the membrane permeability through inhibiting lipid peroxidation and preventing glutathione depletion.

The over production of NO in response to parasitic infection may be considered as one of the risk factors to induce oxidative stress and inflect tissue damage [41]. Finally, NO production correlates positively with tissue fibrosis through inducing fibrogenic cytokines and increasing collagen synthesis [42].

The activities of antioxidant enzymes SOD and CAT in the skin tissue of infected mice with cutaneous leishmaniosis also decreased, where CAT detoxifies hydrogen to water while SOD catalyzes the reduction of superoxide anions to hydrogen peroxide. Herein, treatment with Ag-NPs green synthesized with *M. oleifera* leaf extract may protect cells from damage, demise and dysfunction that caused by cutaneous leishmaniosis-induced oxidative stress. The present results obtained for antioxidant enzyme activities are in accordance to several authors who found significant reduction in GPx in skin of infected mice and attributed these changes to the number of deleterious effects due to the accumulation of superoxide radicals and hydrogen peroxide [43]. 

To explore the mechanism of *M. oleifera* on the attenuation of CL–induced apoptosis, the level of Bax and Bcl–2 proteins were measured in skin homogenates. ROS have been shown to increase the permeability of the mitochondrial membrane and result in mitochondrial failure [39]. The permeability of the mitochondrial membrane is dependent upon the mitochondrial permeability transition pore results in the liberation of cytochrome *c* from the mitochondria to the cytoplasm [39]. Once released, cytochrome *c* can bind to apoptotic protease-activating factor–1(Apaf–1) in the cytoplasm forming a complex that can activate caspase–9 with subsequent activation of death-inducing [44]. In current study, we observed that CL–induced apoptosis in the skin of mice. Our findings showed that mice treated with Ag-NPs green synthesized with *M. oleifera* leaf extract were reversed the alternations of Bcl–2 and Bax levels induced by CL and substantially restored the ratio of Bcl–2/Bax. In the current experiment, *M. oleifera* inhibited all toxic events induced by CL. It is known that herbal scavenges oxygen and nitrogen-based reactants generated in mitochondria, stabilizes mitochondrial membrane and enhances anti-apoptotic signaling.

## 5. Conclusions

Our findings suggested that biosynthesized Ag-NPs from *Moringa oleifera* extract may be involved in preparing a new potential chemotherapeutic agent with very safe and faster in treatment of Leishmaniasis and complete healing after 21 days than standard drug pentostam (that needs more than 28 days for healing) and may be is a possible new drug with clinical efficacy against *L. major*. However, the specific mechanism involved and the optimal safe and effective dose still needs to be determined since the safety of Ag-NPs in human is not determined due to the limited number of well-controlled studies on the potential toxicities of Ag-NPs. However, Kim et al. found no genotoxicity after the oral administration of Ag-NPs of 60 nm average size for 28 days [45] and Lansdown showed that Ag-NPs was not cause neurotoxicity [46]. Furthermore, more studies on the stability of Ag-NPs biosynthesized by *Moringa oleifera* leaf extract should be done in the future to avoid Ag-NPs undesirable changes in size and morphology.

## Figures and Tables

**Table 1:** Experimental determinations of total phenolic and flavonoids contents and antioxidant capacity assays (ABTS, DPPH and FRAB) for *Moringa oleifera* extract.

Parameters	Mean ± SD
Total phenols (mg eq. Gallic acid/g sample)	9.466 ± 0.754
Total flavonoids (mg eq. Rutin/g sample)	0.609 ± 0.026
DPPH (%)	36.88 ± 0.99
ABTS (μmol eq. Trolox/g sample)	4.989 ± 0.034
FRAB (μmol eq. Trolox/g sample)	0.185 ± 0.0005
